# The Trajectories and Correlation between Physical Limitation and Depression in Elderly Residents of Beijing, 1992–2009

**DOI:** 10.1371/journal.pone.0042999

**Published:** 2012-08-15

**Authors:** Xia Li, Wei Wang, Qi Gao, Lijuan Wu, Yanxia Luo, Zhe Tang, Xiuhua Guo

**Affiliations:** 1 Department of Epidemiology and Health Statistics, School of Public Health and Family Medicine, Capital Medical University, Beijing, China; 2 Beijing Key Laboratory of Clinical Epidemiology, Beijing, China; 3 Department of Epidemiology and Social Medicine, Xuanwu Hospital, Capital Medical University, Beijing, China; 4 Beijing Geriatric Clinical and Research Center, Beijing, China; Federal University of Rio de Janeiro, Brazil

## Abstract

**Background:**

Physical limitation and psychological distress have been reported to be related, but studies describing the change of instrumental activities of daily living (IADLs) and depression syndrome over time or exploring the link pattern for their development are limited. The study was to assess distinctive patterns for the development of physical limitation and depression and to explore their correlation to form a proper prevention strategy.

**Methods:**

Dual trajectory analysis was conducted using data from the Beijing Longitudinal Study of Aging (BLSA) 1992–2009 hosted by Xuanwu hospital for subjects with full information on depression and physical limitation for all available visits. Physical limitation was measured by the Instrumental Activities of Daily Living (IADL) scale and depression by the Center for Epidemiological Studies Depression scale (CES-D). The covariates were gender, age at baseline and number of chronic conditions.

**Results:**

Three heterogeneous trajectories for physical limitation and two distinct groups for an increase in depression were detected. Among them, 10.13% of subjects experienced an increase in physical limitation, while 13.22% demonstrated a high, stable level of depressive mood. In all, 80.4% of the subjects enjoyed a relatively low, stable level of IADL and CES-D scores. People in the late increase group for IADL score were more likely to have depressive mood when adjusted for gender, age and number of chronic conditions (OR = 3.900, 95%CI = 1.347–11.290).

**Conclusions:**

The development of physical limitation among the elderly may significantly increase the risk for depressive symptoms.

## Introduction

Depression in the elderly population has been a major public health issue. Depression mainly affects those with chronic medical illnesses or cognitive impairments; causes suffering, family disruption, and disability; worsens the outcomes of many medical illnesses; and increases mortality [1]. Using the Geriatric Depression Scale (GDS) as a measurement instrument, over 20% of community- dwelling elderly people in China have been reported to suffer from symptoms of depression [2]–[6]. Data from the Hypertension in the Very Elderly Trial revealed a strong connection between depression and subsequent mortality, cardiovascular morbidity and incident dementia [7]. The Medical Outcomes Study reported that depression associated with impairment and disability is equal to that attributable to cardiovascular disease and greater than that due to other chronic physical disorders, such as hypertension, diabetes, and arthritis [8]. People with depression symptoms demonstrated a greater use of all healthcare services with the exception of preventive medicine services [9]. Depression also delayed the process for rehabilitation from coronary heart disease [10], which indicated that more medical resources were consumed without efficiency.

Physical functional status, or the capacity to perform daily activities, has always been the focus in gerontology. The “activities of daily living” (ADLs) index tends to decrease in late life [11], which serves as a threat to one’s capability for social participation and living safely and independently [12], thus becoming one of the stressors for depression [13]. Depression has been found to be significantly associated with long-lasting decrements in functioning that are comparable to, or even greater than, those associated with chronic medical illnesses [14].

Several studies have been made to explore the impact of depression for functional impairment in post-stroke patients [15] or post-myocardial infarction patients [16]. A number of experimental studies and several observational studies have also investigated the relationship between physical health status and depressive symptoms, using samples drawn from clinically depressed patients [17] or from persons not clinically depressed 18,19. Although the findings are not entirely consistent, studies have shown that ADL disability is consistently a powerful predictor of depression in the elderly [20]. Effects of ADL limitations on depression might be much stronger than influences of depression on ADL limitations [21].

The development of physical limitation was mainly investigated by scholars in sociology. Socioeconomic status [22], social network [23] and health behavior [24] might account for the development of physical limitation. However, for community residents, the development of physical limitation, as measured by the ADL was diverse and highly dynamic [1], [25]. Our study was to provide a better understanding for the phenomenon and to explore its underlying mechanisms in a Chinese population.

## Results

### The Baseline Characteristic for Subjects

As shown in Table 1, at baseline, the mean age of the 316 subjects was 63.54 (SD 6.21), with a range of 55–83 years. Among these subjects, 59.2% was female, 44.6% was illiterate, 33.2% completed primary school education, 9.2% completed junior high school education, 5.1% graduated from senior high school, and 7.9% had completed college education or above.

**Table 1:** Characteristics and IADL and CES-D score for subjects.

Index		Total	Male	Female	Statistic	*P* value
IADL#	1992	6.31±1.20	6.28±1.17	6.33±1.23	−0.466	0.642
	1994&	6.15±0.72	6.22±0.97	6.09±0.47	1.372	0.172
	1997&	6.44±1.57	6.57±1.96	6.35±1.23	0.977	0.330
	2000	6.10±0.80	6.05±0.29	6.13±1.01	−0.600	0.549
	2004	6.47±1.84	6.48±1.90	6.47±1.81	0.760	0.939
	2007	6.70±2.24	6.49±1.73	6.83±2.51	−0.955	0.341
	2009	6.86±2.73	6.76±2.48	6.92±2.88	−0.266	0.791
CESD*	1994	7.00 (4.00–12.00)	7.00 (4.00–11.50)	7.00 (4.00–12.00)	−0.256	0.798
	1997	11.00 (5.00–16.00)	11.00 (5.00–16.00)	10.00 (6.00–16.00)	−0.297	0.766
	2000	6.00 (3.00–12.00)	5.00 (3.00–10.00)	7.00 (3.00–12.00)	−2.582	0.010
	2004	7.00 (3.00–13.75)	6.00 (3.00–13.00)	7.00 (3.00–14.00)	−0.622	0.534
Age at baseline@		63.54±6.21	64.47±6.84	62.90±5.66	2.146	0.033
Chronic conditionŝ		0.00 (0.00–1.00)	0.00 (0.00–1.00)	0.00 (0.00–1.00)	−0.406	0.684
Education%	College or above	25 (7.9)	17 (13.2)	8 (4.3)	40.265	<0.001
	Senior high school	16 (5.1)	5 (3.9)	11 (5.9)		
	Junior high school	29 (9.2)	17 (13.2)	12 (6.4)		
	Primary school	105 (33.2)	58 (45.0)	47 (25.1)		
	Illiteracy	141 (44.6)	32 (24.8)	109 (58.3)		

# IADL was assessed in the years 1992, 1994, 1997, 2000, 2004, 2007 and 2009; two independent t test was used for log10 transformation, 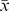
±SD.

& t’ test was used in the year of 1994 and 1997;

* CESD was measured in the years 1994, 1997, 2000 and 2004, Mann-Whitney U test was used; Median (P_25_–P_75_).

@t’ test was used, 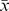
±SD.

% Chi-square was used.

∧ Mann-Whitney U test was used.

### Score of ADL and CES-D in Every Wave

The ADL and CES-D scores in every wave and the comparison between male and female were listed in Table 1. The gender difference in ADL and CES-D score was not statistically significant except in CES-D in the year of 2000. Therefore, the dual trajectory analysis was not used separately for males and females.

### Trajectories for IADL Limitation

**Figure 1:**
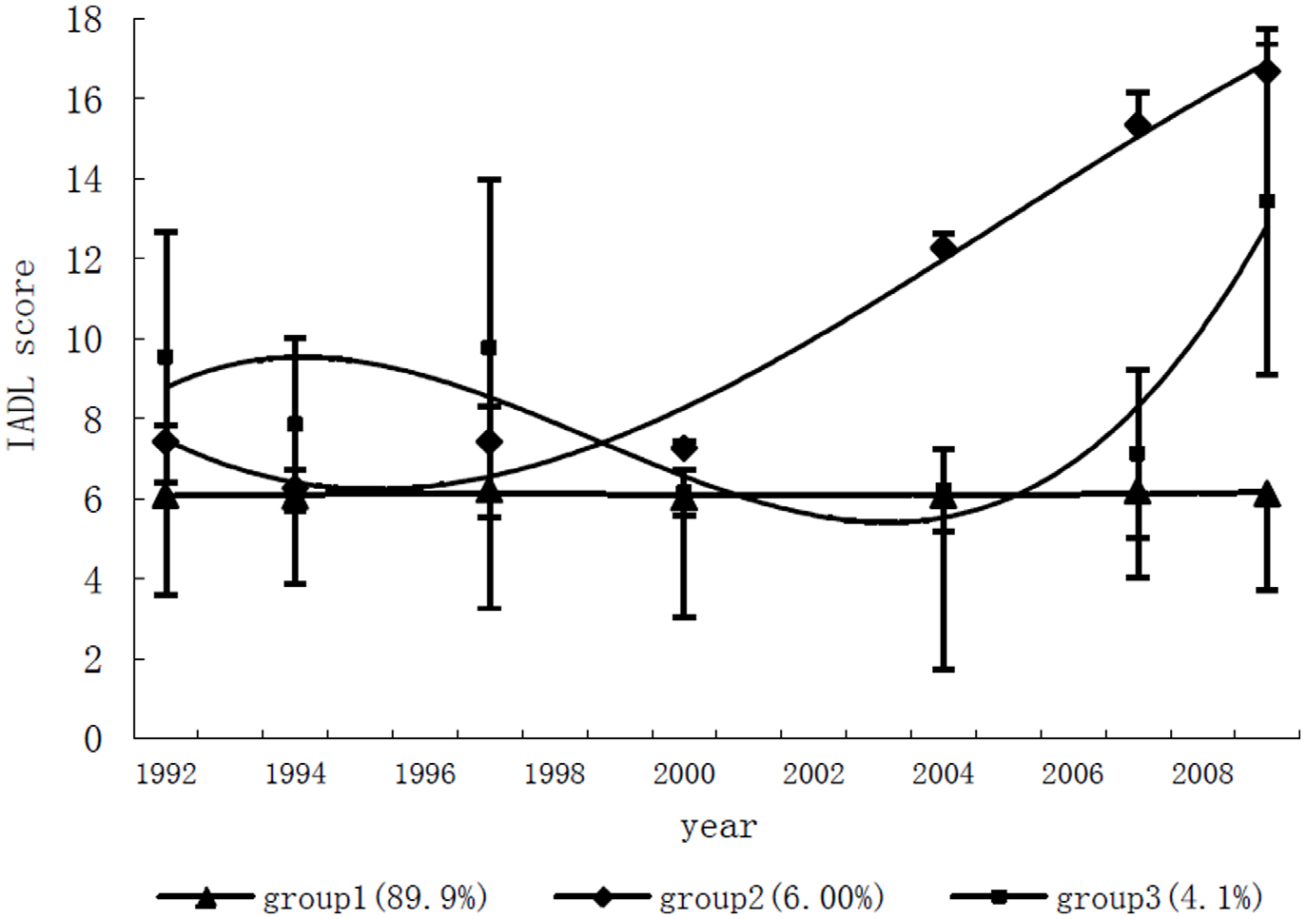
Trajectories of IADL score over time. Bar represented the SD for IADL score in each group in every visit.

A three-group model was selected for the lowest BIC of −3034.14, with 4.1% of the subjects in the smallest trajectory group. The three groups in Figure 1 were named after the trends as follows: “No Increase” (group 1), “Late Increase” (group 2), and “Decrease-Increase” (group 3). The percentage and average posterior probability for trajectory assignment in each subgroup were shown in Table 2. The majority was in the No Increase group (89.9%) with the IADL score around 6 points indicating no major physical limitation in the 17 years. Six percent of the subjects were in Late Increase group. The IADL score started at 8 points and increased sharply since 1999 to around 16 points. In the Decrease-Increase group, 4.1% of subjects have an IADL score that decreased slightly in the first eight years then increased to 12 points. The average posterior probability for group assignment in each group was no less than 0.96, indicating a low rate (less than 0.04) for misclassification.

**Table 2:** Percentages of the sample assigned to each trajectory group and average posterior probability for trajectory assignment.

	N (%)	Average posterior probability for group assignment
IADL		
No Increase (Group1)	284 (89.87)	0.99
Late Increase (Group2)	19 (6.01)	0.96
Decrease-Increase (Group3)	13 (4.12)	0.97
CES-D		
Variation at low level (group 1)	275 (87.02)	0.98
Stable high (group 2)	41 (12.98)	0.93

### Trajectories for Depression

**Figure 2:**
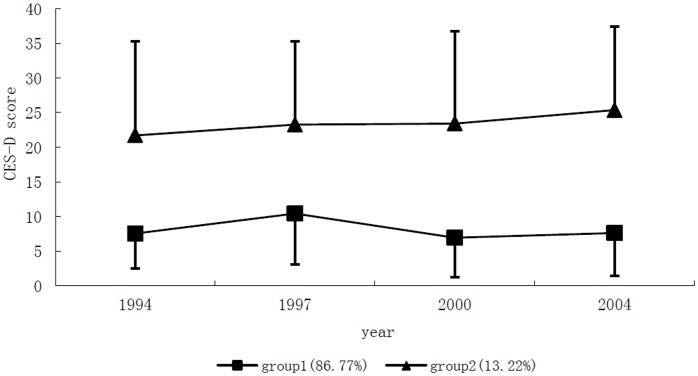
Trajectories of CES-D score over time. Bar represented the SD for CES-D score in each group in every visit.

A two-group model was selected for the lowest BIC of −4451.85, with 12.6% of the subjects in the smallest trajectory group (refer to the supplement). The two groups in Figure 2 were named after the trends as follows: “Variation at Low Level” (group 1) and “Stable High” (group 2). The percentage and average posterior probability for trajectory assignment in each group were shown in Table 2. Among them, 87.0% of the subjects were in group 1, with a CES-D score around 23 points. On the other hand, 13.0% of the subjects were in group 2, with a CES-D score around 5 to 10 points. The average posterior probability for group assignment in both groups was no less than 0.93, showing a low rate (less than 0.07) for misclassification.

### Correlation in the Trajectories of Physical Limitation and Depression

The joint trajectory group membership probability was shown in Table 3. Among them, 80.4% of the subjects were in the group 1 for CES-D scores and in the group 1 for IADL scores; 9.5% in the group 2 for CES-D scores and in group 1 for IADL scores; 4.1% in group 1 for CES-D scores and in group 2 for IADL scores; 1.9% in group 2 for CES-D scores and in group 2 for IADL scores; 3.2% in group 1 for CES-D scores and in group 3 for IADL scores; 0.9% in the group 2 for CES-D scores and in group 3 for ADL scores. The Spearman correlation coefficient was 0.159, but with statistical significance (*p* = 0.005).

**Table 3:** Joint trajectory group membership probability of IADL score and CES-D score (%).

	CES-D	
IADL Score	Variation at low level (Group 1) N (%)	Stable high (Group 2) N (%)
No Increase (Group1)	254 (80.4)	30 (9.5)
Late Increase (Group2)	13 (4.1)	6 (1.9)
Decrease-Increase (Group3)	10 (3.2)	3 (0.9)

A logistic regression analysis was used to further detect predictors for the adverse CES-D group. Gender, age at baseline, and number of chronic disease were considered as potential confounding variables for the IADL trajectory group that were forced into the model (Table 4). As it revealed, when gender, age and number of chronic conditions were adjusted, IADL trajectory group was an independent risk factor for the adverse CES-D trajectory group. Subjects from the Late Increase group in IADL scores were more likely found in the adverse CES-D group, with ORs of 3.900 (95%CI: 1.347–11.290).

**Table 4:** Logistic regression for adverse CESD group membership.

	B	SE	Wald	*P*	OR	95% CI for OR	
						Lower	Upper
Constant	−4.864	1.991	5.971	0.015	0.008		
Gender	0.559	0.377	2.190	0.139	1.748	0.834	3.663
Age at baseline	0.027	0.028	0.928	0.335	1.027	0.973	1.085
Number of chronic disease#			1.086	0.581			
1 condition	0.330	0.377	0.766	0.381	1.391	0.665	2.909
≥2 conditions	−0.187	0.591	0.100	0.752	0.829	0.261	2.641
IADL trajectory group*			7.247	0.027			
IADL trajectory group 2	1.361	0.542	6.299	0.012	3.900	1.347	11.290
IADL trajectory group 3	0.908	0.711	1.633	0.201	2.480	0.616	9.987

* With group 1 as reference.

# With none condition as reference.

## Discussion

Depression and physical limitation were both major issue of public health in elderly people [1], [11]. In order to understand the potential linkage between the development of disability and depression, the dual-trajectory analysis was used. Group-based models (GBMs) [26] have become increasingly popular alternative methods for longitudinal data analyses. They assumed that the study population was made up of a finite number of sub-populations defined by distinctive patterns of growth. The analysis aimed to identify these sub-groups or latent classes, as well as to assess their association with individual-level covariates. Unlike random-effects growth curve modeling, GBM is person or subject-centered (not variable-centered), and group-based (not individual-based) akin to cluster analysis for longitudinal data. The trajectory analysis was a technology in GBM and was used in the study of physical aggression [27], obesity [28] and anxiety [29]. The dual model jointly estimated the trajectories of two distinct but related longitudinal outcome series and analyzes the connection between the two outcome series, which might evolve contemporaneously (co-morbidity) or evolve over different time periods (heterotypic continuity, or temporal interdependence [30]. This longitudinal association could also be assessed in conventional growth curve modeling; however, it could only estimate the overall association between the two outcomes calculated over heterogeneous subpopulations. The underlying group heterogeneities were “averaged out” in this procedure.

There are few published reports describing the trajectory of ADL or depression. An analysis in China (from the Chinese Longitudinal Healthy Longevity Survey 1998–2005) using the zero-inflation Poisson model for group-based analysis found three trajectories for disability. In that report, 61.3% of the subjects were in the “Low-Stable” group, 26.45% in the “Low-Rapid Increase” group, and 12.3% in the “High-Stable” group. Females and senior people were more likely to be found in the “Low-Rapid Increase” and “High-Stable” disability groups. Non-agricultural elderly people were likely to be in the “Low-Rapid Increase” and “High-Stable” groups [25]. In comparison, the percentage of subjects in the “No Increase” group was higher in our analysis. This could be partially explained by the construction of the sample. The prior study involved elderly people aged over 80 years old and surveyed in 22 provinces in China. As these subjects differed by socio-economic status, it would be expected that more changes in disability patterns might be observed. However, they might be limited by the four waves. A report from Taiwan using latent growth curve analysis indicated a rising trajectory of depressive symptoms in both Taiwanese elderly males and females over 10 years. The trajectory of depressive symptoms in males was affected by perceived health and disability and the trajectory of depressive symptoms in females was influenced by disability and social support [31]. In our study, the IADL and CES-D scores were only slightly different in males and females at each wave. As subjects were limited in the current study, the gender-specific trajectory analysis was not performed, and gender was not a significant predictor (OR = 1.748, 95%CI = 0.834–3.663) for the adverse CES-D group.

The association between depression and disability has not been consistently reported. A report from nursing home patients showed that the presence of depression and/or anxiety has a statistically and clinically significant negative impact on well-being but not on disability [32]. Further, reports from this cohort study [33], [34] and EURODEP [35] indicated a positive association between the depression and physical limitation, which is in accordance with this analysis. Several reports further analyzed the relationship between depression and disability. The onset of disability was reported to have stronger effects on change in CES-D scores than recovery from disability. These effects also differed by type of transition in disability statuses [36]. Results from the Cardiovascular Health Study suggested that persistently elevated depressive symptoms in elderly persons are associated with a steep trajectory of worsening functional disability [37]. The association between disability and depression was further separated into three components: a strong contemporaneous effect of change in disability on depressive symptoms, a weaker 1-year lag effect of change in depressive symptoms on disability, and a weak correlation between the trait components of depression and disability [38]. Consistent with these findings, the association between the “Late Increase” group in IADL was associated with increased depression symptom reporting. However, in our analysis, their cause-effect relationship could not be identified.

This study was limited by the assessment of depressive symptoms and ADL disability. Only 316 persons completed the IADL examination and depression symptom report in the years 1994, 1997, 2000 and 2004 that was used in this analysis. To establish patterns for IADL and CES-D development, all possible assessment results were used to establish the model and posed a difficulty in understanding the results from logistic regression at the same time. Further analysis for latent growth curve analysis might be used for more informative results. Another limitation was the statistical analysis procedure itself. Only 316 persons were in this study, causing difficulty for extrapolation. Those who died or were lost from the cohort may be the one with severe physical and mental distress [20]. In our study, the lost ones were with similar condition for chronic disease (t = 0.467, p = 0.641), but higher IADL score (t = 8.347, p<0.001) and CES-D score (t = 2.318, p = 0.21). Joint modeling that took both the longitudinal analysis and survival analysis into account might be used in future.

Regardless of the factors that need further investigation, the identification of the trajectory for depressive symptoms as well as disability in activities of daily living is very important. Given the important impact of activities of daily living functioning on utilization of medical services and quality of life, prevention or reduction of depressive symptoms should be considered as an important reason for intervention.

## Methods

### Participants

Data were derived from the Beijing Longitudinal Study of Aging (BLSA), a community-based prospective cohort study hosted by Xuanwu hospital. The procedures for sampling and data collection were described in detail elsewhere [39]. Briefly, in August 1992, a random sample of 3257 community dwellers aged 55 years or above from three districts (Xuanwu district in an urban area, Daxing district in a suburban area and Huiruo district in a rural and near mountains) were recruited. The sample was stratified for gender and age group (starting from 55 years old, at 5-year intervals). The distribution of educational background for the final sample was consistent with that of aging population in Beijing obtained from the Fourth National Census Data of China. In the years 1994, 1997, 2000, 2004, 2007 and 2009, all survivors of this cohort were contacted for re-examination. Among them, 316 subjects who completed the ADL examination and depression symptom report in the years 1994, 1997, 2000 and 2004 visits were engaged in this analysis.

### Measurements of Physical Limitation

Physical limitation was assessed by the 12-item Activities of Daily Living scale. Examples of ADL items are “Can you, fully independently, dress yourself?” “... go up and down the stairs?” “... prepare dinner?” “... get in and out of a bed?” “... do the grocery shopping?” and“... take a bath or shower?”. Each item has three response categories (1 =  I can do that easily and without help; 2 =  I can do that with some help; 3 = I can do that only with help from others). It covered the ability to perform self-care, such as feeding, grooming, dressing, bathing, moving around inside home, cooking, managing money, taking a bus, shopping, walking 300 meters, and walking up and down stairs. The last six items were identified as the Instrumental Activities of Daily Living (IADL), which was reported more likely to impaired than Basic Activities of Daily living (BADL) [40]. The IADL scores for the years 1992, 1994, 1997, 2000, 2004, 2007 and 2009 were used for analysis.

### Measurements of Depression

The Center for Epidemiological Studies Depression scale (CES-D), a 20-item self-reporting questionnaire, was used to assess depression symptom. Total scores ranging from 0 to 60, reflected the frequency with which a given symptom was experienced during the previous week. The more severe depressive symptoms corresponded to a higher score. In this study, the standard cut-off of 16 points was used as the measure of depression. The CES-D scores for the years 1994, 1997, 2000 and 2004 were used for analysis.

### Measurements of Other Covariates

The analysis took a number of possible confounders into account in the relationship between ADL disability and subsequent depressive symptoms. Socio-demographic variables, such as age at baseline and gender were served as covariates in the analyses. Chronic conditions were ascertained from a self-report of a physicians’ diagnosis, including arthritis, cancer, cardiovascular disease(hypertension, angina, heart infarction, arrhythmia, rheumatic heart disease), stroke(including transient ischemia attack), diabetes, or respiratory system disease (chronic bronchitis, emphysema, asthma, tuberculosis). The number of chronic conditions was used as dummy variable in the analysis (0 = none, 1 = 1 chronic condition; 2 = equal or more than 2 chronic conditions).

### Statistical Analysis

Trajectory modeling is a kind of group-based model [26], which identifies homogeneous groups within a population assumed to contain different course of development. To examine patterns linking IADL disabilities and depression syndrome over time, the dual trajectory model, a more advanced version of group-based modeling was adopted. The dual model analyzed the connection between two outcome series, which might develop contemporaneously, or develop over different time periods (heterotypic continuity, or temporal interdependence), while determining the linkage between two series as a function of individual characteristics and other covariates. The trajectory of IADL limitation and depression, reported at waves 2 to 4, was in a censored normal distribution. The Bayesian Information Criteria (BIC) was used to identify the optimal number of homogenous groups, with an additional requirement that the smaller group consist of at least 5% of the sample. Dual trajectory model analysis is conducted using the SAS procedure “PROC TRAJ” (version 9) developed by Jones & Nagin [41]. Two-independent t-tests or the Mann-Whitney U test was used to compare the IADL and CES-D scores in each year, comparing male and female subjects. Binary logistic regression was used to explore the risk factor for the adverse CES-D trajectory group. Gender and age at baseline, as potential confounding factors, were factored into the model. The IADL trajectory group was used in dummy variables, with group 1 (No Increase) as the reference. SAS software version 9.1.3 was used for all the analyses (Statistical Analysis System Inc, 2006). All probability values presented were for two-tailed tests. P<0.05 was considered to indicate statistical significance.

### Ethical Approval

All participants were asked to sign an informed consent form and the project was approved by ethic committees of Xuanwu Hospital Capital Medical University. Written informed consents were obtained for every subject.
